# Aboveground Allometric Models for Freeze-Affected Black Mangroves (*Avicennia germinans*): Equations for a Climate Sensitive Mangrove-Marsh Ecotone

**DOI:** 10.1371/journal.pone.0099604

**Published:** 2014-06-27

**Authors:** Michael J. Osland, Richard H. Day, Jack C. Larriviere, Andrew S. From

**Affiliations:** 1 U.S. Geological Survey, National Wetlands Research Center, Lafayette, Louisiana, United States of America; 2 Five Rivers Services, LLC, U.S. Geological Survey, National Wetlands Research Center, Lafayette, Louisiana, United States of America; DOE Pacific Northwest National Laboratory, United States of America

## Abstract

Across the globe, species distributions are changing in response to climate change and land use change. In parts of the southeastern United States, climate change is expected to result in the poleward range expansion of black mangroves (*Avicennia germinans*) at the expense of some salt marsh vegetation. The morphology of *A. germinans* at its northern range limit is more shrub-like than in tropical climes in part due to the aboveground structural damage and vigorous multi-stem regrowth triggered by extreme winter temperatures. In this study, we developed aboveground allometric equations for freeze-affected black mangroves which can be used to quantify: (1) total aboveground biomass; (2) leaf biomass; (3) stem plus branch biomass; and (4) leaf area. Plant volume (i.e., a combination of crown area and plant height) was selected as the optimal predictor of the four response variables. We expect that our simple measurements and equations can be adapted for use in other mangrove ecosystems located in abiotic settings that result in mangrove individuals with dwarf or shrub-like morphologies including oligotrophic and arid environments. Many important ecological functions and services are affected by changes in coastal wetland plant community structure and productivity including carbon storage, nutrient cycling, coastal protection, recreation, fish and avian habitat, and ecosystem response to sea level rise and extreme climatic events. Coastal scientists in the southeastern United States can use the identified allometric equations, in combination with easily obtained and non-destructive plant volume measurements, to better quantify and monitor ecological change within the dynamic, climate sensitive, and highly-productive mangrove-marsh ecotone.

## Introduction

In response to climate change and land use change, species distributions are changing across the globe [1]–[3]. Some of these distributional changes are resulting in comparatively large ecological transformations, especially where traditional ecosystem boundaries (i.e., ecotones) are migrating [4]–[6]. In areas with shifting ecotones, ecologists and environmental managers seek information that will enable them to better understand the ecological implications of ecosystem transformations.

In coastal wetland ecosystems, abrupt ecotones are common and occur across multiple abiotic gradients (e.g., inundation, salinity, and macroclimatic gradients) [7]–[10]. Since tidal wetland ecotones involve foundation plant species, sensu [11]–[14], with divergent growth forms and functionality (e.g., graminoid plants, microbial mats, succulent plants, and woody mangrove plants), the ecological implications of shifting ecotones in coastal wetlands can be substantial.

In the southeastern United States and other tropical-to-temperate climatic transition zones, the mangrove-salt marsh ecotone is dynamic, visually striking, and highly productive [15], [16]. Mangroves are woody foundation plant species that are sensitive to extreme winter temperatures and dominant in warmer climates [17], [18]. In contrast, salt marsh graminoids and salt marsh succulent plant foundation species are dominant in cooler coastal reaches [8], [19] where extreme winter temperature events lead to mangrove mortality and/or limit mangrove forest development, reproduction, and dispersal [10], [20], [21]. At the poleward mangrove-marsh ecotone, mangrove abundance and coverage is winter temperature-sensitive in that it increases and decreases in response to the absence or presence of extreme winter events, respectively [22]–[24]. In response to changing climatic conditions, a decrease in the frequency, duration, and/or intensity of extreme winter temperatures is expected which would facilitate poleward mangrove range expansion at the expense of salt marsh vegetation [10]. In the southeastern U.S., the northward expansion of mangroves is expected to occur in Texas, Louisiana, and parts of Florida.

The ecological implications of mangrove expansion are poorly understood. Mangroves and salt marsh ecosystems provide many important ecosystem goods and services including fish and wildlife habitat, carbon storage, nutrient and sediment retention, coastal protection, and maintenance of coastal food webs and fisheries [25]. Although salt marsh and mangrove forest ecosystems are both highly valuable and have many ecosystem goods and services in common, there are structural and functional differences between these two ecosystems as well as differences in the provision of some ecosystem goods and services. Like many other ecotones, structural and functional complexity is higher at the mangrove-marsh ecotone than in adjacent graminoid or mangrove-dominated ecosystems. As a result, it is likely that ecological resilience and the supply of some ecosystem goods and services within the mangrove-marsh ecotone is equivalent to or even greater than in adjacent marsh or mangrove-dominated areas. However, our understanding of the ecosystem goods and services that would be affected by mangrove expansion and replacement of salt marsh is limited, and coastal wetland ecologists seek information and tools that will enable them to better quantify ecological changes associated with mangrove expansion at the expense of salt marshes [26]–[31]. Here, we provide equations that can be used to quantify changes in important aspects of aboveground mangrove structure near and within the dynamic mangrove-marsh ecotone.

**Figure 1:**
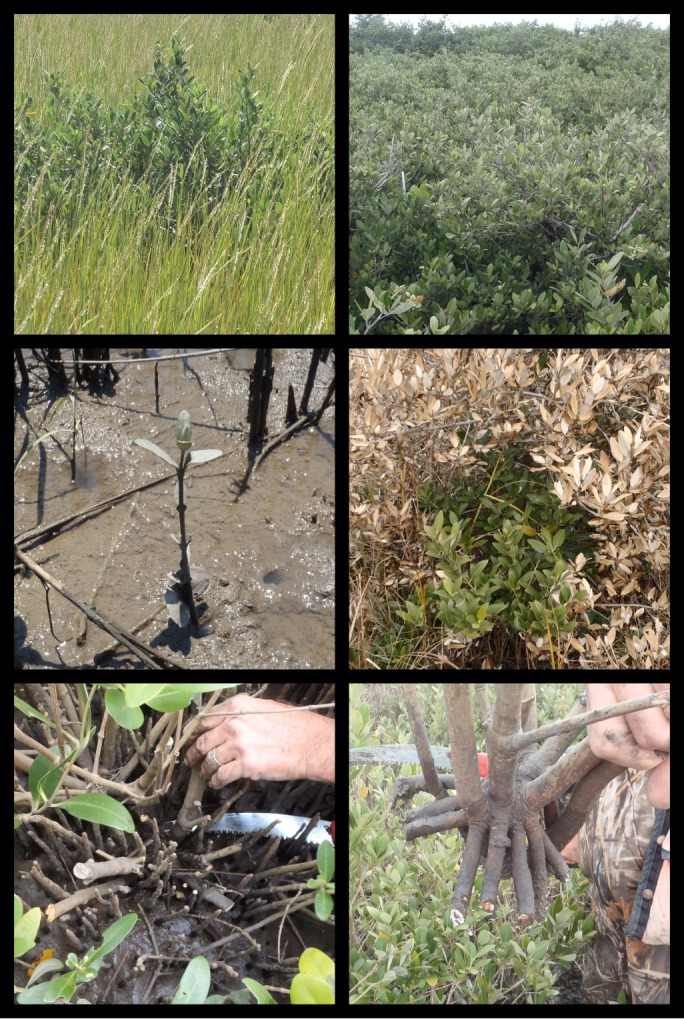
Photos of freeze-affected black mangroves (*Avicennia germinans*) in Louisiana (USA) near their northern range limit. The upper two photos show the shrub-like morphology. The middle left photo shows the size of the smallest individuals included in the analyses. The middle right photo shows leaf damage from an extreme winter temperature event in January, 2014. The lower two photos show the high stem density of freeze-affected individuals.

Aboveground biomass is an important and fundamental metric for quantifying change in coastal wetland ecosystems. Many important coastal wetland ecological functions and services are affected by changes in aboveground biomass, structure, and productivity, including resilience to sea level rise [32]–[34], carbon storage [18], [35]–[37], coastal protection [18], [38], [39], recreation, and fish and avian habitat [40], [41]. Allometric equations provide an approach for estimating plant biomass and structural attributes via easily-obtained and non-destructive measurements. Most allometric models for determining mangrove aboveground biomass have been developed in tropical climates in the absence of extreme freeze events [42]. Within the freeze-dependent mangrove-marsh ecotone, traditional metrics for quantifying mangrove forest aboveground biomass are not appropriate because the morphology and architecture of freeze-affected mangrove trees is different than tropical mangrove trees. In the absence of freeze events and where freshwater and nutrient resources are abundant, tropical mangrove plants can develop into comparatively tall and straight trees. In contrast, at the poleward range limit of mangroves, severe freeze events can either lead to total mangrove mortality or cause aboveground structural damage that is followed by vigorous basal resprouting (Figure 1). As a result, mangroves in freeze-prone areas are often shorter, wider, multi-stemmed, and more shrub-like relative to their tropical counterparts growing in resource-rich environments, but see [43]. Tree-focused allometric models for quantifying aboveground biomass (i.e., dbh and/or height-based models) [42], [44]–[49] are not suitable for freeze-affected and shrubby mangrove individuals.

Our objective was to develop aboveground allometric models for freeze-affected black mangroves (*Avicennia germinans*). In the southeastern United States, *A. germinans* is the most cold-tolerant mangrove species and the most abundant mangrove species at the northern range limit of mangrove forests. Based upon studies from other shrub-dominated ecosystems [50]–[53] and models for stunted mangroves in New Zealand [54], southern Florida (USA) [55], [56], and Iran [57], we expected that crown area and height measurements could be used to develop allometric models for quantifying total aboveground biomass, leaf area, and leaf biomass for freeze-stunted *A. germinans* individuals.

## Materials and Methods

### Study Area

**Figure 2:**
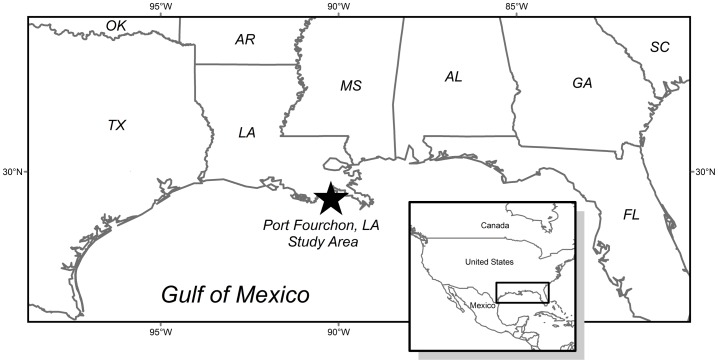
Map highlighting the mangrove-marsh ecotone where this study was conducted (Port Fourchon, Louisiana [USA]).

Allometric equations were developed using mangroves from two tidal wetland sites near Port Fourchon, Louisiana (USA; 29°8′52″N, −90°14′38″W and 29°6′58″N, −90°11′26″W′; Figure 2), which is within the Mississippi River Deltaic Plain. Tidal wetlands in this area are abundant and dominated primarily by *Spartina alterniflora* (smooth cordgrass) and/or *A. germinans*. For more information about the study area, see [26], [29], [58]–[62]. Port Fourchon is near the northern limit of *A. germinans* along the Gulf of Mexico coast and is periodically exposed to extreme winter events that can damage or kill *A. germinans* (see photos in Figure 1), reducing areal coverage and limiting structural development. Historical accounts of *A. germinans* in the region document freeze-induced mortality during the 1960's [63] and 1980's [24]. The aerial extent and abundance of *A. germinans* has expanded and contracted in response to extreme winter events [22], [24], [63], [64]. Although most of the *A. germinans* stands in the Port Fourchon vicinity were less than 3 m in height at the time of this study, some individuals were between 4.5 and 5 m in height and some stands were in the 3–4 m height range (RHD and MJO, personal observation). This study was conducted, with permission, on private lands owned by The Louisiana Land and Exploration Company LLC (LL&E) and managed by the Conoco Phillips Company/LL&E office in Houma, Louisiana.

### Allometric Measurements

In February and May 2013, we selected a total of 56 *A. germinans* individuals for allometric equation development. We selected 40 individuals in February and 16 individuals in May. For each individual, we measured the height and the basal diameter at 30 cm above the soil surface. We also measured crown diameter in two perpendicular directions, across the widest crown section (CD_1_) and at the widest section perpendicular to the first section (CD_2_). The two crown diameter measurements were used to calculate crown area via the equation for an ellipse: crown area = [(CD_1_)/2)*(CD_2_)/2)]*π. When an individual had multiple stems, height was measured only on the tallest stem and basal diameter was measured only on the thickest stem. Whereas height was measured to the base of the highest leaf, crown diameter measurements were made to the edge of the most horizontally-distant leaf tips. Crown area and height were integrated into a single estimate of volume via the following equation: volume = crown area*height. Once *in situ* measurements were recorded, we cut each individual at the soil surface and transported the samples back to the laboratories of the U.S. Geological Survey's National Wetlands Research Center in Lafayette, Louisiana.

In the laboratory, the February and May samples were processed in a slightly different fashion since the May samples were collected explicitly for the development of leaf area and leaf biomass allometric equations. Individuals collected in February were dried to a constant mass at 60°C to determine total aboveground biomass (i.e., leaves were not separated from stems and branches prior to drying and weighing). In contrast, the May samples were separated into leaves and non-leaves (i.e., stem+branch) prior to drying, and the area of fresh leaves was determined using a Li-3000C (Licor, Inc., Lincoln, NE, USA). The separated leaf and non-leaf samples were then dried to a constant mass at 60°C to determine leaf biomass, stem+branch biomass, and total aboveground biomass. The data for this study can be found in Dataset S1.

### Data Analyses

Linear regression models were developed for the following response variables: (1) total aboveground biomass; (2) leaf biomass; (3) stem plus branch biomass; and (4) leaf area. The independent variables (i.e., crown area, height, volume, and basal diameter) were evaluated alone and in combination via simple and multiple regression analyses. Response and independent variables were natural log transformed prior to analysis. For each equation, we calculated a correction factor (CF) sensu Sprugel [65], where CF = exp (SEE^2^/2) and where SEE is the Standard Error of the Estimate. These correction factors are to be applied to the back-transformed estimates of the response variables in order to correct for a small underestimation associated with using log transformed data in the regression analyses [50], [66]. For example, for an allometric equation of the form ln (y) = *a*+*b* * ln (x), y should be calculated as follows: y = CF * (exp [*a*+*b* * ln (x)]). Data analyses were conducted in SAS Version 9.3 (SAS Institute, Cary, NC, USA) and Sigma Plot Version 12.5 (Systat Software, Inc., San Jose, CA, USA).

## Results

**Figure 3:**
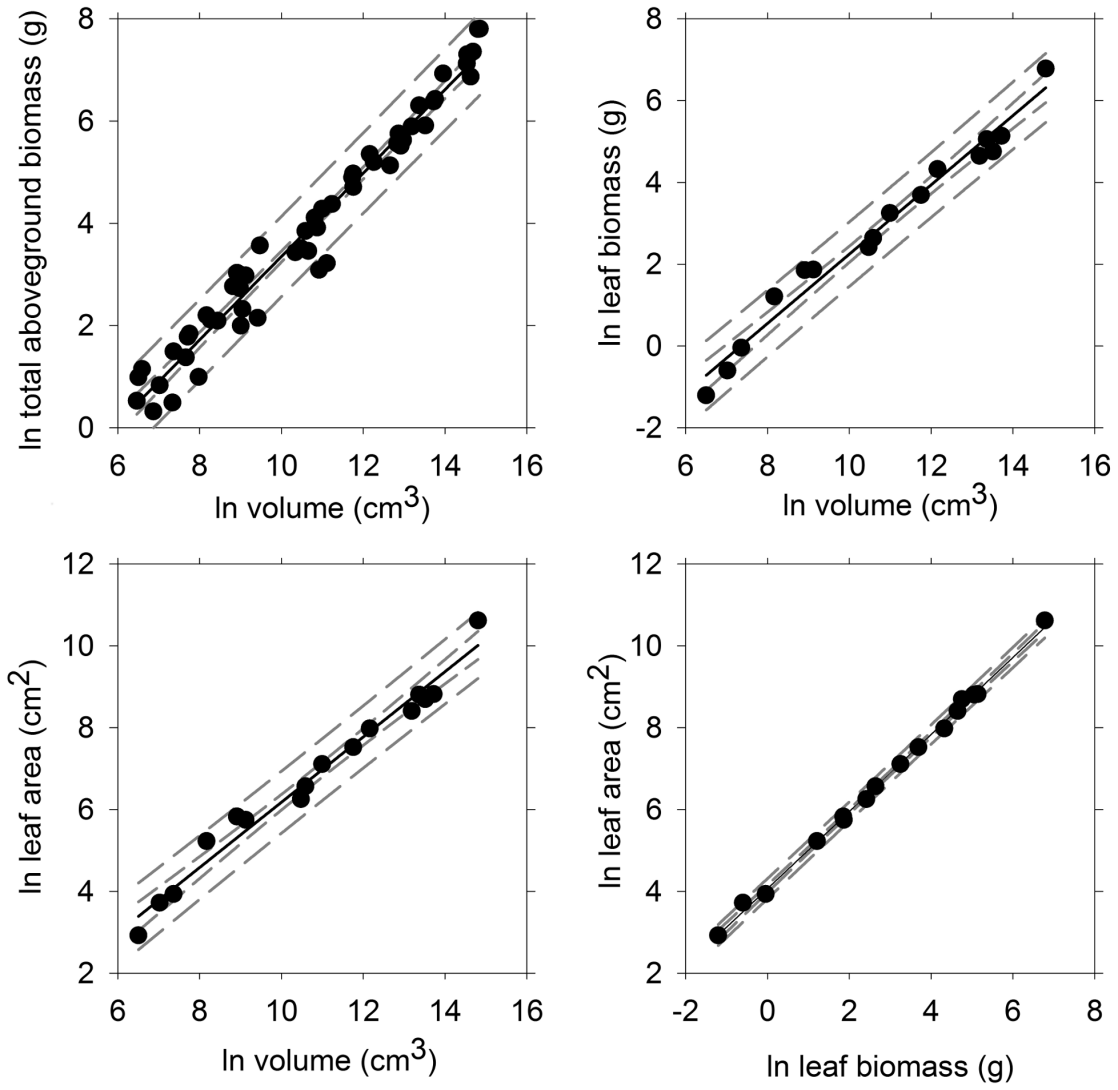
Aboveground allometric relationships for freeze-affected black mangrove (*Avicennia germinans*) individuals. The short and long dashed lines show the 95% confidence and prediction bands, respectively. Note the natural log scale on both axes.

We measured *A. germinans* individuals within a height range of 31–157 cm, a crown diameter range of 4–180 cm, and an aboveground biomass range of 1–2,452 g (Table 1). For the smallest plants, the measurements of crown diameter were essentially the lengths of two pairs of leaves on a single un-branched stem. In contrast, the largest plants contained multiple stems and branches extending to almost 1 m from a central base (Figure 1). We developed and compared multiple allometric equations using various combinations of predictor and response variables resulting in the selection of those shown in Table 2 and Table S1. The range limits presented in Table 1 identify boundaries for the application of the developed equations and should be used to avoid unwarranted extrapolation. Plant volume was selected as the best predictor of all of the response variables (i.e., leaf area, leaf biomass, stem plus branch biomass, and total above ground biomass) (Table 2, Figure 3, Table S1). Additional models based upon other combinations of measurements were also significant (Table S1). These additional models may be useful for researchers that want to determine biomass using alternative combinations of variables.

**Table 1:** Sample size and measurement range of the variables used to develop allometric models for freeze-affected black mangroves (*Avicennia germinans*).

Variable	Sample Size	Minimum	Maximum
Height (cm)	56	31	157
Basal Diameter at 30 cm (mm)	56	1	31
Crown Diameter (cm)	56	4	180
Crown Area (cm^2^)	56	19	23,629
Volume (cm^3^)	56	641	2,811,792
Total Aboveground Biomass (g)	56	1	2,452
Leaf Biomass (g)	16	0.3	881
Stem and Branch Biomass (g)	16	2	1,556
Leaf Area (cm^2^)	16	19	40,941

**Table 2:** Selected allometric equations for freeze-affected black mangrove (*Avicennia germinans*) individuals.

Response (y)	Predictor (x)	*a* (SE)	*b* (SE)	Adj-R^2^	RMSE	CF	d.f.
Total Aboveground Biomass	Volume	−4.8045 (0.2250)	0.8157 (0.0204)	0.97	0.39	1.0781	56
Leaf Biomass	Volume	−6.2219 (0.3834)	0.8468 (0.0348)	0.98	0.36	1.0654	16
Stem plus Branch Biomass	Volume	−4.5075 (0.2971)	0.7682 (0.0269)	0.98	0.28	1.0388	16
Leaf Area	Volume	−1.8036 (0.3681)	0.7981 (0.0334)	0.97	0.34	1.0602	16
Leaf Area	Leaf Biomass	4.0626 (0.0437)	0.9418 (0.0121)	0.99	0.11	1.0057	16

These are all equations of the following form: ln(y) = *a*+b*ln(x). CF is the correction factor sensu Sprugel [65]. Additional equations can be found in Table S1.

## Discussion

In this study, our primary objective was to develop aboveground allometric equations for freeze-affected *A. germinans* individuals near and within the poleward mangrove-marsh ecotone in the southeastern United States. Allometric equations enable scientists to estimate plant community structural attributes via easily-obtained and non-destructive measurements. Although aboveground allometric models have been developed for mangrove species in different abiotic settings [42], most of these models are for trees growing in tropical wet climates. At the poleward range limit of mangrove forests, extreme winter temperature events affect the morphology of mangrove plants and result in plants that sometimes resemble shrubs (i.e., short, wide, and multi-stemmed individuals) rather than trees. To our knowledge, our study is the first to develop allometric models for freeze-affected and shrub-like *A. germinans* individuals at their northern range limit.

Mangrove shrub or dwarf morphologies can result from various abiotic conditions, including drought stress, freeze stress, salinity stress, nutrient limitation, and hydrologic isolation [44], [67]–[71]. Shrub-based aboveground allometric models have been developed for some mangroves in some of these abiotic conditions. In New Zealand, near the southern latitudinal limit of mangroves and in an area with shrubby mangroves less than 1 m tall, Woodroffe [54] developed allometric equations that predict *A. marina* biomass using mean canopy width measurements. In an arid high-latitude mangrove forest in Iran, Parvaresh et al. [57] developed allometric equations that predict *A. marina* biomass from measurements of crown diameter. In a south Florida (USA) dwarf mangrove forest located in an irregularly-inundated basin setting, Ross et al. [55] developed allometric equations that predict *A. germinans*, *Rhizophora mangle*, and *Laguncularia racemosa* biomass from measurements of stem basal diameter and crown volume. In another south Florida dwarf mangrove forest, Coronado-Molina et al. [56] developed allometric equations that predict *Rhizophora mangle* biomass from measurements of height, crown diameter, and the number of prop roots. Collectively, these four studies along with our results show that crown area-focused measurements, sometimes in combination with other measurements (e.g., plant height, number of prop roots), can be used to develop allometric equations for estimating aboveground biomass of mangrove individuals with dwarf or shrub morphologies.

How do we envision that these equations will be used? In response to future changing winter temperature regimes and accelerated sea level rise, black mangroves are expected to migrate both poleward [10] and landward [72] along the northern Gulf of Mexico coast, in many cases at the expense of salt marshes, tidal freshwater systems, and/or upland forests. At many of these ecotones, ecologists will be challenged to characterize and monitor the structural changes that are already occurring or are expected to occur in the future. To the north and south of these ecotones (i.e., in salt marshes and large mangrove forests, respectively), methods for characterizing aboveground biomass and vegetation structure are well-established. Vegetation methods used in salt marshes are quite different than those used in mangrove forests. For example, whereas ground-layer measurements and vegetation samples (e.g., biomass clip plots and stem measurements from plants less than 2 m in height) are typically taken in small plots (∼1-m^2^) within marshes, tree-focused measurements (e.g., dbh and/or tree heights) are typically taken in large plots (∼100-m^2^) within tropical mangrove forests. Vegetation measurements in the freeze-affected and structurally-complex mangrove-marsh ecotone require a hybrid sampling approach that includes measurements tailored for the stunted morphology of freeze-affected mangroves (e.g., crown area and plant height measurements) as well as the multiple other vegetation strata that could be present ranging from graminoid and succulent marsh plants to large (i.e., 2–10 m tall) mangrove forest trees. See Osland et al. [36] for an example of the multiple plot sizes and stratum-specific measurements required to sample marshes and mangroves within a single study design. Sampling designs within mangrove-marsh ecotones often need to be customized to fit the range of plant species and morphologies present. Where conditions allow multi-stemmed shrubby mangroves to grow into large multi-stemmed trees, traditional tree-focused metrics can be applied to each individual stem contained within a tree [43].

Our findings and allometric equations provide a foundation for characterizing aboveground structural changes of freeze-affected black mangroves within mangrove-marsh ecotones. In combination with a robust sampling design, these equations can be used to quantify the temporal and spatial changes in plant community structure in response to the presence or lack of extreme climatic events including freeze events, drought, and hurricanes, as well as slower abiotic changes associated with sea level rise. Plant community structural information can be used to investigate the effects of ecosystem transformations and ecotone migration upon important ecosystem functions and services (e.g., sediment trapping, soil elevation change, nutrient cycling, fish and wildlife abundances, wave attenuation, carbon storage). When paired with carbon concentration data and information regarding belowground processes and soil carbon accumulation, our equations can provide a foundation for quantifying the aboveground carbon present in freeze-affected black mangrove forests which is information that can contribute to estimates of carbon sequestration and inform best practices for carbon management and climate change mitigation [35], [37]. The biomass equations can also be used to evaluate plant community development and functional equivalency after wetland restoration and/or management. The leaf area equations may be incorporated into ecophysiological and/or biogeochemical models, for example [60], [73].

In summary, we developed aboveground allometric equations for freeze-affected black mangroves (*A. germinans*) which can be used to quantify the following: (1) total aboveground biomass; (2) leaf biomass; (3) stem plus branch biomass; and (4) leaf area. Plant volume (i.e., a combination of crown area and plant height), was selected as the optimal predictor of these response variables. Coastal wetland scientists in the southeastern United States can use these equations to better quantify and monitor ecological changes within the dynamic and climate sensitive mangrove-marsh ecotone.

## Supporting Information

Table S1 **Additional allometric regression equations for freeze-affected black mangrove (** ***Avicennia germinans*** **) individuals.**
(DOC)

Dataset S1 **The data used to develop allometric equations for freeze-affected black mangrove (** ***Avicennia germinans*** **) individuals.** An excel file that has two spreadsheets: (1) a data spreadsheet (spreadsheet entitled “data”); and (2) a spreadsheet that includes a description of column names and units contained within the data spreadsheet (spreadsheet entitled “legend”).
(XLSX)
