# Supplementary material for: Aboveground Allometric Models for Freeze-Affected Black Mangroves (Avicennia germinans): Equations for a Climate Sensitive Mangrove-Marsh Ecotone
Source: PLoS One. 2014 Jun 27;9(6):e99604. doi: 10.1371/journal.pone.0099604 (PMC4074035; doi:10.1371/journal.pone.0099604)
Supplement: Table S1 — Additional allometric regression equations for freeze-affected black mangrove ( Avicennia germinans ) individuals. (DOC) [file pone.0099604.s001.doc]

**Table S1. Additional allometric regression equations for freeze-affected black mangrove (*Avicennia germinans*)** individuals.

| Response (y) | Predictor(s) (x_1_, x_2_, x_3_) | a (SE) | b (SE) | c (SE) | d (SE) | Adj-R^2^ | RMSE | CF | d.f. |
| --- | --- | --- | --- | --- | --- | --- | --- | --- | --- |
| Total Aboveground Biomass | Area, Ht, Diam | -4.5344 (1.0865) | 0.6785 (0.0703) | 0.7567 (0.3605) | 0.4326 (0.2557) | 0.97 | 0.38 | 1.0741 | 56 |
| Total Aboveground Biomass | Area, Ht | -5.8373 (0.7799) | 0.7322 (0.0638) | 1.1798 (0.2642) | NA | 0.97 | 0.38 | 1.0768 | 56 |
| Total Aboveground Biomass | Area, Diam | -2.2813 (0.1735) | 0.7195 (0.0696) | 0.8049 (0.1900) | NA | 0.97 | 0.39 | 1.0790 | 56 |
| Total Aboveground Biomass | Area | -2.4356 (0.1944) | 0.9945 (0.0289) | NA | NA | 0.96 | 0.45 | 1.1051 | 56 |
| Total Aboveground Biomass | Ht | -13.1805 (0.8255) | 3.9734 (0.1903) | NA | NA | 0.89 | 0.71 | 1.2879 | 56 |
| Total Aboveground Biomass | Diam | -1.3267 (0.2526) | 2.6355 (0.1182) | NA | NA | 0.90 | 0.67 | 1.2523 | 56 |
| Total Aboveground Biomass | Volume, Diam | -4.3234 (0.3075) | 0.6847 (0.0624) | 0.4619 (0.2089) | NA | 0.97 | 0.38 | 1.0726 | 56 |
| Leaf Area | Area | 0.6214 (0.3184) | 0.9591 (0.0472) | NA | NA | 0.96 | 0.40 | 1.0836 | 16 |
| Leaf Biomass | Area | -3.6482 (0.3346) | 1.0175 (0.0496) | NA | NA | 0.97 | 0.42 | 1.0927 | 16 |
| Stem and Branch Biomass | Area | -2.1764 (0.2664) | 0.9236 (0.0395) | NA | NA | 0.97 | 0.34 | 1.0578 | 16 |

These are all equations of the following form: ln(y) = a + b*ln(x_1_) + c*ln(x_2_) + d*ln(x_3_). CF is the correction factor sensu Sprugel **.** Additional equations can be found in Table 2.
